# Understanding Combined Health and Business Risk Behaviour: Small Tourism Firm Owners Reopening Amid COVID-19 in Pingyao, China

**DOI:** 10.3390/bs12100358

**Published:** 2022-09-26

**Authors:** Haizhou Zhang, Zhaoyuan Shi, Jieqi Chen, Ziang Zhang

**Affiliations:** 1 Geography Research Unit, University of Oulu, 90014 Oulu, Finland; 2 School of Geography and Planning, Sun Yat-sen University, Guangzhou 510275, China; 3 School of Geography and Tourism, Anhui Normal University, Wuhu 241002, China; 4 School of Geography, Nanjing Normal University, Nanjing 210023, China

**Keywords:** small tourism firm, reopen business, risk decision, protection motivation theory, theory of planned behaviour, Ancient City of Pingyao (ACPY)

## Abstract

This study explores the psychological factors affecting small tourism firm (STF) owners’ decision making about reopening businesses in the midst of COVID-19 based on protection motivation theory and the theory of planned behaviour. The data were collected from a sample of 300 STFs in the Ancient City of Pingyao when the lockdown policy was lifted in China. A symmetric approach, i.e., partial least squares structural equation modelling (PLS–SEM), and an asymmetric model, i.e., a fuzzy set/qualitative comparative analysis (fsQCA), were used to analyse the net effect of the psychological determinants and correlations between the variables leading to high and low behavioural intentions to reopen businesses. The results indicate that social norms and perceived business uncertainty were the critical factors influencing the intention to reopen. The pathway (low perceived risk of infection, low perceived business uncertainty, high reward, high response efficacy, high self-efficacy, high attitude, and high subjective norm) was only one configuration for a high intention to reopen. The study results are discussed based on dual-process theory, and practical implications are offered to guide STF recovery amid COVID-19.

## 1. Introduction

Public health events, as an increasingly common form of risk/crisis, have numerous unprecedented and serious impacts on the tourism sector, particularly in regard to the current COVID-19 pandemic [1,2]. What is worse is that small tourism firms (STFs), as the main body of tourism in underdeveloped countries, are more vulnerable due to a lack of preparedness and the absence of resources needed to counter negative impacts [2] STFs are often run by members of a single family or have only one owner without sufficient funding or professional information on navigating risks and crises [3,4], let alone this unprecedented long-lasting crisis [5]. Amidst the current situation, in which risk remains omnipresent, STF recovery and sustainability are important problems worldwide [1].

The period from lockdown to recovery is an emerging but important scenario in public health events [6,7], marked by the conclusion of early turbulent chaos but the continuation of small-scale outbreaks and viral mutation. High tourist mobility leads to the risk that tourist destinations may be closed again at any time if imported infected cases result in a re-emergence of the epidemic. STF owners must urgently choose whether to return to work. In one respect, reopening businesses helps to decrease economic losses. However, unlike non- or low-human-contact industries, tourism is characterised as highly mobile with high levels of interpersonal contact, meaning that reopening combines the risks associated with owner-embodied health levels and business uncertainties [8].

Regarding the academic research on tourism risk and crisis management, in contrast to the abundance of tourism crisis/disaster management studies focussing on governments, tourist destinations, and multinational corporations, the more vulnerable STFs are underrepresented [9]. On one hand, the current COVID-19 epidemic-induced social and economic results differentiate the pandemic from spatiotemporally small-scale epidemics [10,11], postgeological disasters [12,13], and human-caused social crises [14,15,16] upon which existing recovery research focusses. On the other hand, reopening business, a more complex and conflicting decision, cannot be explained well by the existing tourist risk behaviour or health prevention behaviour research [17]. Thus, this research attempts to capture a unique period by conducting a survey with STF owners amid COVID-19 in the Ancient City of Pingyao (ACPY), aiming to (a) propose a psychological model that can interpret the behaviour of business reopening amidst COVID-19, (b) analyse the relationship between embodied and business risk factors and behavioural intentions, and (c) identify the causal mechanisms for high and low behavioural intentions. This study not only contributes as a knowledge reference for practical tourism recovery but also, more importantly, expands the understanding of the decision-making risk behaviour types in the field of tourism risk and crisis management.

## 2. Theoretical Background

### 2.1. Theoretical Foundations: Towards a Psychology of STF Owners’ Behaviours

Unlike large tourism companies, STF owners must make the reopening decision individually, without feasibility studies or mathematical simulations and predictions [18,19]. As STF owners are both the decision makers and the implementers of the reopening plan, stressful conditions combining both the infection threat and unsustainable business operations particularly matter [20,21]. STF reopening cannot be viewed as a pure business decision rooted in economic rationalism. In contrast, according to dual-process theory, seriously stressful conditions interfere with rational and deliberative processes, leading decision-makers to fall back on more intuitive, automatic processes, a relatively faster, more superficial, and spontaneous mode based on intuitive associations [22]. Therefore, the basis of commonly used theories in risk and crisis management studies [17]—such as the resource-based view [23], socioemotional wealth theory [24], etc.—is not fully applicable to STF reopening behaviour.

**Figure 1:**
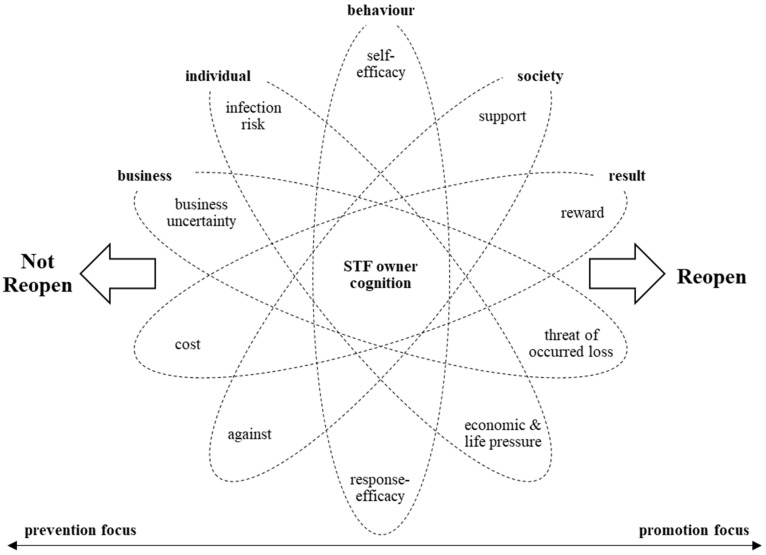
Schematic representation of STF owners’ business decision to reopen. Source: Adapted from [31,32].

We first adopted the reductionist perspective to identify the key components of this complex phenomenon (see Figure 1). Shutdowns during the outbreak caused major business financial losses and might have even threatened firm survival. Although timely reopening entails the possible rewards of earning profits, the business risk cannot be ignored. Uncertainty during this stage also involves commercial risks and new costs associated with reopening. Individually, most owners with stress disorders also take physical health conditions seriously. Reopening involves more frontline exposure, producing occupational risks and even some disagreement from individual social networks. Overall, STF owners’ decision processes cannot be encompassed by a business economic algorithm or a complex rational reasoning outcome [25]. Under the risk of stress arising from social, psychological, and physiological aspects, the STF owners’ decision outcomes can be understood as more intuitive outcomes [26]. Problem-solving decisions in psychology are not viewed as simple cognitive processes based on calm and rational logic but on heuristics and emotions, providing an opportunity to gain a much clearer understanding of the causes of predictable behaviour [27,28]. Based on social psychological epistemology, the reopening decision is an individual one that owners make based on their understanding of human–firm–environment interactions, informing an integrated understanding and awareness. Therefore, psychological cognition is introduced as a theoretical foundation to capture the specific attributes of the business decision to reopen, providing a theoretical supplement to the existing risk and crisis management studies. Accordingly, the conceptual model of this study was developed based on the sociopsychological theoretical frameworks PMT [29,30] and TPB [3].

#### 2.1.1. Protection Motivation Theory (PMT)

To better understand how individuals cope with threats, Rogers proposed PMT, arguing that individual behaviour in response to a potential threat is not a straightforward outcome of the physiological stress response but is the result of a cognitive mediating process in response to an evaluation of the threat and coping levels [29,33]. The cognitive process results in effects from fear-arousing communications about threats, whereupon the process mediates individual attitudes. By affecting a person’s attitude, the process induces changes in subsequent behaviours. Thus, each component of the fear appeal is responsible for a specific cognitive mediating process, which leads to protection motivation. Through a set of psychological constructs and communication tools, PMT helps reveal the main elements responsible for attitude change. Its strong explanatory and predictive power has been verified for preventive and protective behaviours in specific potential threat scenarios, including preventive behaviours in response to COVID-19 [34] and various tourism risk contexts [35].

#### 2.1.2. The Theory of Planned Behaviour (TPB)

Ajzen developed the TPB considering nonvolitional factors as behavioural determinants to explore a more realistic context in which the subjective probabilities of success and actual control are less than perfect [3]. According to the TPB, behavioural intention is a function of attitude, subjective norms, and perceived behavioural control. As one of the most predictive attitude–behaviour theories, the TPB has been widely used to interpret different behaviours in both the risk context and the tourism field [36,37], including travel behaviours in the post-COVID-19 pandemic era [38].

### 2.2. Integrated Model and Hypotheses

Although these two theories have been widely used to predict diverse risk and tourist behaviours, they have had limited predictive power in some research contexts because of their specific mechanisms and limitations [39]. For instance, in addition to behavioural ability, PMT considers the variable of behavioural influence from perceived threats, while the TPB focusses more on people’s attitudes and norms regarding behaviour. Taylor et al. stated that a single theory is insufficient and that integrating distinctive constructs from competing theories into one or more poly-theoretical models can fully illuminate and more effectively predict individual behaviour [40]. Thus, a model integrating PMT and the TPB has been adopted by scholars to increase the predictive power [41,42]. However, this model is absent from tourism risk studies. We used the integrated model, which includes the main cognitive aspects illustrated in Figure 1. The dependent variable is the behavioural intention, taken as a proxy measure of the likely decision to reopen. After clarifying this feature of PMT and the TPB, some relationships that were hypothesised and tested are presented below.

According to PMT, the coping behavioural intention is positively associated with the threat appraisal process. Reopening is one such process that carries threats of both infection risk and business uncertainties. For individuals, infection risk and fear discourage the reopening decision. Wang et al. reported that the prerequisite for industry resumption is the guarantee of no human infection [35]. The same reasoning applies to tourists. Because of the pandemic, tourists’ risk perceptions, fear, reduced free time, and disposable incomes all hinder tourist behaviour [43]. In addition, maintaining social distancing means a potentially low frequency of business transactions in indoor STFs [44]. Coupled with the instability of the epidemic, uncertainties are significant to businesses during this stage [45]. Bernanke’s real option theory observes that the irreversibility of many decisions and possible sunk costs cause enterprises to opt to withdraw from decision making [46], as many large tourism enterprises in China do, such as Shanghai Disneyland and Happy Valley Theme Park. Therefore, consistent with PMT and the dimensions of the individual and firm in the owner’s decision framework, we present the following hypothesis:

**H1.**  *Perceived infection risk (PIR) has a negative effect on the behavioural intention to reopen (BIR)*.

**H2.**  *Perceived business uncertainty (PBU) has a negative effect on BIR*.

Reward refers to the subjective or objective sense of benefit that an individual perceives will result from the behaviour. In PMT, the construction is suggested to be considered a component of the behavioural decision, as the individual will not take action if the perceived benefit is less than the loss [29]. In the current study, home quarantine can reduce the likelihood of damage from threats, but owners will reopen if the perceived reward is greater. Kahneman and Tversky’s prospect theory notes that people tend to be risk seeking when determining losses, and higher perceived rewards result in a stronger gambling psychology [47]. Wang even found that many people cannot parse the risk probabilities if the rewards are sufficiently high [48]. In addition, Morrish and Jones found that tourism entrepreneurs who successfully recovered generally accepted higher-than-normal risks in the postdisaster recovery in New Zealand [31]. Xu’s survey also notes the risk preference of small and medium-sized enterprise business management during the COVID-19 pandemic [21]. Thus, the following hypothesis is proposed:

**H3.**  *Reward (Rew) has a positive effect on BIR*.

The coping appraisal process is the individual assessment of the ability to cope with and avoid threats, including response efficacy, self-efficacy, and cost [49]. Response efficacy refers to compliance with reopening as an effective mechanism for eliminating the threat. Self-efficacy refers to the perceived types of skills and measures needed for reopening, which are linked to an individual’s capabilities. Response cost refers to perceived opportunity costs in terms of the money, time, and effort required to reopen. Those who have the requisite knowledge about the effectiveness of the coping mechanism and who possess higher levels of self-efficacy regarding protection behaviour are more likely to adopt adaptive behaviours [3,50]. The same rationale applies to STF owners’ reopening [31]. Response cost is the converse of the reward function. That is, if the cost is sufficiently high, individuals will be reluctant to adopt the behaviour, and vice versa [25]. The coping process aligns with the two behavioural dimensions and results in the owner’s decision framework. Hence, the following hypotheses are proposed:

**H4.**  *Response efficacy (RE) has a positive effect on BIR*.

**H5.**  *Cost (Cos) has a negative effect on BIR*.

**H6.**  *Self-efficacy (SE) has a positive effect on BIR*.

According to Ajzen [3] and Francis et al. [51], attitude is an individual’s overall evaluation of the focal behaviour. Subjective no24rms are an individual’s perception of important others’ thoughts and views about a target behaviour—i.e., the individual’s own estimate of the social pressure to perform the behaviour. In the current study, attitude refers to owners’ overall feelings about reopening, while subjective norms are defined as their perceived social pressure to reopen. The TPB indicates that a more positive attitude results in a greater perceived social pressure promoting the behaviour and hence a stronger behavioural intention. STF owners have several attitudes due to differences in the aspects of personality traits, resources, ability to integrate information, etc. Owners with pessimistic and negative attitudes tend to think that resuming operations is futile and meaningless; thus, they are less likely to do so. Regarding social influence, although they are independent firm decision-makers, owners rely on information and judgements by authoritative government officials and experts because owners’ capabilities are limited [2,52]. Moreover, since STF development is characterised by geographical concentration, peer actions and knowledge sharing within the cluster are important factors influencing owners’ decision making [19]. Asgary et al. found that social values and family constraints were the key influencing factors in the STF recovery process in Pakistan’s postflood period [4]. Based on this analysis, the following hypotheses are proposed:

**H7.**  *Attitude (Att) has a positive effect on BIR*.

**H8.**  *Subjective norms (SN) have a positive effect on BIR*.

## 3. Methodology

### 3.1. Measurement Instruments

A compatible measurement for the new issue is lacking. Based on interviews and related existing studies, this study developed scales measuring STF owners’ behavioural intentions to reopen. Through a snowball approach, we interviewed ten owners, two administrative staff members, and one resident of a tourist community (see Table 1 for their profiles). The sample includes all STF business types in the case destination. As the researcher is a native of the area and the interviews were conducted during the outbreak, the informants were her acquaintances or were introduced through them. The semistructured interviews mainly pertained to people’s knowledge and attitudes towards the COVID-19 outbreak and the influence of the pandemic on STF businesses and their reopening. The core interview questions aimed to inform the contextual interpretation of the PMT and TPB constructs. Then, the interview coding results and study research results were used as references to develop scales that are in line with the TACT principle (target, action, context, and time) [51].

Based on Fisher et al. [53], Wolff et al. [54], and Wang et al. [35], we used both perceived vulnerability and perceived severity to measure PIR. The PBU, PR, RE, and RC scales were developed based on the interview coding results and qualitative research [31,32]. In addition, the statements and forms of each item were designed by comprehensively referencing the PMT construct in different studies. For instance, to measure PBU, the respondents were asked to indicate the extent to which they agreed with the two items of business uncertainty after reopening. The scale forms were adapted from Quintal et al. [36]. Two items are the uncertain market and new possible difficulties, which were the most frequent codes relating to PBU in the interviews. Likewise, PR was measured using a two-item scale modified from Wang et al. [35], including the opportunity to make money and the sense of security for STFs. We drew from Fisher et al. [53] and Wang et al. [42] to measure RE with a two-item scale including balancing the previous loss and resolving the backlog. RC was measured with a three-item scale including the extra burden of anti-epidemic measures, energy costs, and financial costs.

SE, Att, SN, and BI were measured using a scale adapted from Bagozzi et al. [55] and Francis et al. [51]. The SE scale included three items: self-confidence, ease, and capacity to reopen the business. The Att scale included three items evaluated by means of semantic differential scales, namely, necessary, favourable, and beneficial. The SN scale included four items assessing the perceived pressure from central groups of people, namely, the government, family, peers, and experts. Given the contextual uncertainty, the BIR scale included only two items, namely, their personal willingness and reopening expectation. Because of the epidemic, a small pretest with 23 online questionnaires was conducted on WeChat and via email with known STF owners through the researcher’s network. The final version of the questionnaire was developed following wording adjustments based on feedback from the pilot survey.

### 3.2. Data Collection

On the first two weekends of April 2020, the field survey was distributed in the ACPY, a popular tourist destination gathering many local STFs. The ACPY was closed on an emergency basis, with 36 local people verified infected (by 1 March 2020) in the early outbreak of COVID-19, and reopened on 25 March 2020, when the local outbreak was controlled and policy permitted. However, certain risks and uncertainties remained in the ACPY tourism environment, such as infections continuing to be imported from overseas and areas where the epidemic continued to develop and grow. Thus, this stage acted as a window for tourism recovery, during which local STF businesses reopened at significantly different times. For operability considerations, we used criteria for screening STF samples, namely, that the business had fewer than ten employees, according to the classification standards prescribed by the Chinese government [56] and the owner was personally involved in the business’ practical operations [18,57]. Three hundred questionnaires were distributed under an administrative one-in-one mode, and 283 completed questionnaires were returned. Questionnaires were eliminated if more than 10% of the data were missing or if 10 or more consecutive questions were given the same scores. Thus, 265 valid data points were obtained and used for further analysis (see Table 2 for their profiles).

### 3.3. Data Analysis

SPSS was used for the descriptive statistical analysis and the common method bias test. The skewness and kurtosis of 19 of 23 items were between −1 and +1 (Table 3), supporting the assumption of normality for all except 4 items. An exploratory factor analysis was conducted for all of the items. The results showed 9 factors with eigenvalues greater than 1, explaining 90.72% of the total variance. The indicators of factorability—namely, communality (0.411~0.869), KMO (0.864), and Bartlett’s test (χ^2^ = 3271.450, df = 253, *p* = 0)—were good. The first factor accounted for only 20.121% of the total variance (less than 50%), indicating that the results were not biased by common method variance.

We used partial least squares structural equation modelling (PLS–SEM) to evaluate the construct properties and to test the hypotheses. According to Hair et al. [58,59], PLS–SEM has certain strengths over covariance-based structural equation modelling that make it more suitable for this study. First, the goal of this research is to identify the key psychological drivers behind the reopening intention, not to test a theory. Second, the structural model integrating PMT and the TPB is complex, with many latent and manifest variables. Third, the sample size is small, and the data are not strictly normally distributed. In SmartPLS 3.2.8, the PLS algorithm and bootstrapping with a subsample size of 5000 were used to calculate the quality criteria for evaluating the model’s overall quality and the significance of the path coefficient (β), resulting in a hypothesis examination [60].

In addition, this study adopted a fuzzy-set qualitative comparative analysis (fsQCA) to categorise the antecedents into causal configurations. In contrast to SEM’s variable-based analytical techniques, an fsQCA is a case-oriented technique focussing on set theoretical associations [61]. Stemming from complex theory and following the configuration theory paradigm, the distinctive nature of an fsQCA provides an alternative to a regression-based analysis [62]. Specifically, this approach considers asymmetry, i.e., the relationship between independent and dependent variables does not always meet the analytical needs; equifinality, i.e., multiple pathways and solutions lead to the same outcome; multifinality, i.e., identical conditions can lead or contribute to different outcomes; and conjunctural causation, i.e., combinations of antecedent conditions, causal mechanisms, or configurations, rather than an estimation of independent net effects [63,64]. As holistic knowledge provides targeted, realistic help, mechanisms are more important than ingredients in some empirical studies [65]. As a pragmatic tool allowing the combination of multiple outcome variables into a single desired outcome condition, an fsQCA is utilised to obtain deeper knowledge of the complex association between multiple independent variables and a behavioural intention [66].

In fsQCA 3.0, the compute variable/calibrate mode was used to calibrate the data by transforming the original ordinal or interval variables into fuzzy variables. Three breakpoints for the construct index measured by five-point Likert-type scales were set as “calibrate (x, 1, 3, 5)”, i.e., 5 = full membership, 1 = full nonmembership, and 3 = crossover point [67]. Then, a necessary condition analysis was used to evaluate the effect of the different variables on the outcome and the outcome’s negation (i.e., high BIR and low BIR). A truth table algorithm was utilised to identify configurations sufficient for the outcome based on the comprehensive assessment of complex, parsimonious, and intermediate solutions.

## 4. Results

### 4.1. PLS–SEM Results

#### 4.1.1. Assessment of the Measurement Model

The measurement model in the current study includes nine first-order reflective constructs. The psychometric properties of the reflective measurement model can be evaluated according to the following criteria: indicator reliability, composite reliability, convergent validity (average variance extracted: AVE), and discriminant validity [68]. After we dropped an item from the cost construct with loadings below the recommended threshold of 0.7, the revised scale appeared to be internally consistent and had convergent validity, as all of the other loadings were above 0.708, the composite reliability was above the 0.7 threshold value, and the AVE was above the 0.5 threshold value. In addition, the square root of the AVE was larger than all other cross-correlations, confirming the discriminant validity of the measurement models. Therefore, the measurement model was psychometrically adequate, as all constructs met the criteria [69].

#### 4.1.2. Structural Model Assessment

PMT, TPB, and IM were acceptable and applicable for the analysis, according to the suitable standardised root mean square residual (SRMR) and goodness-of-fit (GoF) (see values in Table 4). In addition, the coefficient of determination (R^2^) and the construct cross-validated redundancy (Q^2^) show that the three models are appropriate for explaining and predicting BIR. The IM proposed in the current research can explain and predict the factors and mechanisms with high relevance. The path significance in each model was measured based on criteria including path coefficients (β) and significance (*p*- and t values) (see values in Table 5). In the PMT model, RE and Cos had no significant effect on BIR. The other factors had significant effects on intention. Regarding the TPB model, all factors had significant effects on intention at a significance level of *p* < 0.001. The IM results were similar in the significant effect but different in the path coefficients of each construct. In IM, SN was identified as the most important factor, with a path coefficient of 0.334 (t = 5.557, *p* = 0.000). PBU was the second most important factor, with a path coefficient of −0.225 (t = 5.064, *p* = 0.000). Rew, SE, and Att had significant effects on intention at a significance level of *p* < 0.01, with path coefficients of 0.145 (t = 3.166, *p* = 0.002), 0.193 (t = 3.468, *p* = 0.002), and 0.183 (t = 2.962, *p* = 0.003), respectively. PIR affected intention only at a significance level of *p* < 0.05, and its path coefficient was −0.095 (t = 2.435, *p* = 0.015). RE and Cos had no significant effects. Therefore, all of the hypotheses except for H4 and H5 (i.e., H1, H2, H3, H6, H7, and H8) were supported by the results.

### 4.2. fsQCA Results

A QCA is an asymmetric technique in which the condition sets leading to an outcome are different from those leading to the negation of that outcome [67]. The two models were tested separately by an fsQCA: high behavioural intention to reopen business (H-BIR) = f (PIR, PBU, Rew, RE, Cos, SE, Att, SN) and low behavioural intention to reopen business (L-BIR) = f (PIR, PBU, Rew, RE, Cos, SE, Att, SN).

#### 4.2.1. Necessary Conditions

NCA was first used to identify single necessary conditions for H-BIR or L-BIR (see the results in Table 6). Based on the integrated SEM, an NCA determines whether eight antecedent conditions (PIR, PBU, Rew, RE, Cos, SE, Att, and SN) are always present (or absent) in all cases where the outcome is present (or absent). According to Ragin [61], “necessary conditions” are judged by a consistency score threshold of 0.9. Regarding H-BIR, the consistency ranged between 0.612 and 0.969. ~PBU, ~PIR, ~Cos, ~Rew, and SE are necessary conditions for H-BIR. For L-BIR, the consistency ranged between 0.212 and 0.928, and ~Rew and ~Att were necessary conditions.

#### 4.2.2. Sufficient Conditions

The fsQCA truth table algorithm was then utilised to analyse the sufficient conditions for H-BIR and L-BIR (see the results in Table 7). Considering the suggestion of Ragin [67], the frequency thresholds were set at 3 for the two outcome conditions, and the consistency thresholds were set at 0.9. The intermediate solution was used, which included only theoretically plausible counterfactuals [70]. In QCA asymmetrical modelling, coverage and consistency are two probabilistic measures used to confirm the calculated mechanisms for sufficient and consistent causal configurations, respectively, resembling the coefficient of determination and the correlation in symmetrical modelling. Based on the recommendation for the minimum acceptable values of coverage (0.2) and consistency (0.9) [67], these configuration results were sufficient to predict high and low behavioural intentions.

#### 4.2.3. Causal Mechanisms for H-BIR

**Figure 2:**
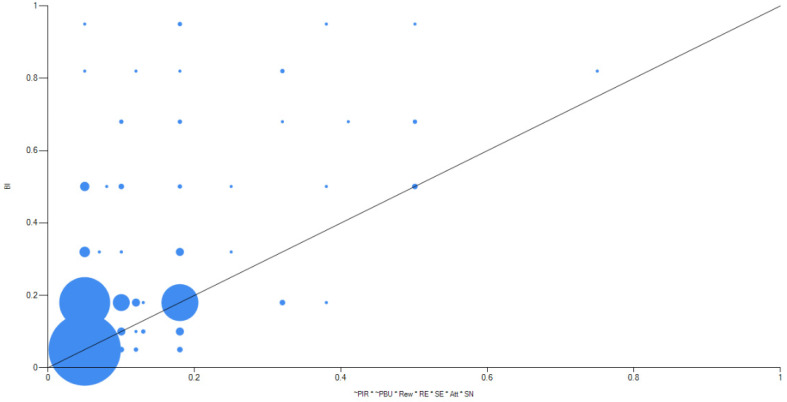
XY plot of M1 for H-BIR.

The results suggest only one pathway, Model_H1_, indicating that the sufficient condition for H-BIR is a combination of low perceived infection risk, low perceived business uncertainty, high reward, high response efficacy, high self-efficacy, high attitude, and high subjective norm. The solution had a high consistency of 0.93 and high coverage of 0.51. As the XY plot of the model in Figure 2 shows, configuration sufficiency was confirmed. That is, the high scores in the causal mechanism led to high scores only in H-BIR, but low scores in the causal mechanism did not lead to specifically high or low scores in the outcome. Thus, the combination of the IM antecedents, except cost, was sufficient to achieve a high behavioural intention.

#### 4.2.4. Causal Mechanisms for L-BIR

In contrast to the above single pathway for H-BIR, the fsQCA results suggest eleven causal mechanisms for L-BIR, proving the asymmetry of the fsQCA method based on set theory. The solution had a high consistency of 0.96 and high coverage of 0.69. The row coverages of M_L7_~M_L11_ were much lower than those of the other six models, and those of M_L10_ and M_L11_ were lower than the threshold value of 0.2. Considering the statistical and practical significance, we mainly analysed the other six models, M_L1_~M_L6_. All six configurations showed that the absence of both Att and SN was always combined with the absence of another condition. In addition, PBU was present in five pathways, and Cos was absent in five other pathways. Thus, perceived business uncertainty, nonattitude, nonsubjective norms and noncost usually lead to low behavioural intention. When we compared the two causal mechanisms for H-BIR and L-BIR, the solution robustness was partly confirmed, as the conditions in M_L1_, M_L3_, and M_L4_ were opposite to those of M_H1._ That is, a combination of conditions led to a high behavioural intention, while a combination of absences for some of these causal conditions led to a low behavioural intention. Additionally, the absence of PIR was significant in both configurations, meaning that PIR was a sufficient condition for reopening intention depending on the specific combination with additional causal conditions. These results confirm the complex nature of STF owners’ decision making regarding reopening, considering the heterogeneous interaction among psychological factors related to facing risk and uncertainty.

## 5. Conclusions

Focussing on STF owners’ reopening decisions amidst COVID-19, this study explores an efficient theoretical understanding of decision-making behaviours combined with both physical health threats and business uncertainty. A psychological model is proposed that integrates PMT and the TPB, and the explanatory power was verified by the data collected from STFs in the ACPY, China. The relationships among multiple factors and the reopening behavioural intention were revealed in the assessment of the structural model. Perceived infection risk and business uncertainty negatively influence the intention; self-efficacy, attitude, and subjectivity positively influence the intention; and response efficacy and cost have no significant effects on the intention. Furthermore, a striking contrast was found between causal mechanisms for high behavioural intention, which had only 1 path, and for causal mechanisms for low behavioural intention, which included 11 paths. This finding validates the owner’s sensitivity to the risky decision-making nature of reopening during COVID-19. Only the psychological combination of a low degree of perceived infection risk and perceived business uncertainty and a high degree of reward, response efficacy, self-efficacy, attitude, and subjective norms contributes to STF owners’ high intentions to reopen during COVID-19.

### 5.1. Discussion

Partly consistent with the existing research on tourism risk [35,71] and commercial crisis management [9,17], the absence of risk and uncertainty is a core necessary condition for a high behavioural intention. However, perceived business uncertainty has a greater impact, indicating that risk perception influences decision making more than does physical health, a finding that cannot be accounted for by prospect theory [47]. As business uncertainty originates from the external environment and directly influences economic results, owners reopen. However, infection risk is simply an increasing possibility due to behaviour. The reflection effect states that the likelihood that people will make risky decisions in loss domains is significantly increased under stress [72]. Similarly, we argue that the preceding business closure and the threatening financial situation created an urgent stress window for STF owners. The acute extrinsic stress altered decision making by modulating risk taking and exacerbating a behavioural bias. In addition, the chaos in the early epidemic stage was effectively controlled by the Chinese government, establishing strong national credibility during epidemic prevention efforts [73]. Social trust and guarantees, for instance, free COVID-19 medical treatment in China, also possibly mitigated the effect of the perception of infection risk on STF owners’ intentions, as risk information originates from the top. In contrast, information regarding business certainty is derived from the bottom of the market, creating owner sensations rather than perceptions. These two conversely originating risks work differently in decision making [74].

Second, in the coping appraisal of PMT, the PLS–SEM results show that response efficacy and cost do not significantly impact behaviour, whereas self-efficacy does. Ifinedo also found that cost does not have a significant impact, and he suggested that this finding is strongly related to context and behaviour [41]. In the present study, it could be interpreted that, in the decision to reopen business, STF owners care less about the costs and results than they do about whether they will succeed as a business. This interpretation would verify the STF reopening decision as having components of adventure and blindness, as found by Xu [21]. It is more consistent with the automatic and impulsive cognition system of dual-process theories, a system that is driven by associations, personal relevance, and situational/contextual information [74]. With limited information and knowledge about the epidemic and market, STF owners can choose to persist in their business only as long as they can. However, the business operational flexibility of STFs cannot be neglected, supporting various innovative ways of conducting business and effectively reducing the possible loss due to uncertainty. For example, some STF owners chose to open periodically and make sales randomly based on actual passenger flows and the daily outbreak situation; some restaurant owners switched from dine-in to take-out businesses; and some homestay hosts began to apply no-touch technology to their business, such as check-ins.

Finally, subjective norms and attitudes are the factors that most strongly influence STF owners’ reopening intentions. The absence of attitude is a core necessary condition for a low behavioural intention. This factor is different from the results of other research using the integrated PMT and TPB model, indicating that reopening amid COVID-19 is also more complex, multidimensional, and socioculturally related than in other direct threat-resistant behaviours, such as vaccination, long-term healthy exercise, and daily habits to avoid the virus [34]. Menard et al. confirmed that an individual’s personal orientation towards collectivism has an impact on psychological ownership and the intention to perform protective behaviours [75]. In this case, Chinese collectivism increased significantly in the anti-COVID-19 period as people adopted a “joint defence and control” social system [73], which played a potential role in Chinese people’s decisions amid COVID-19. As clustered development aligns with the nature of small enterprises [19], both public administrative guiding policy at the macro level and entrepreneurial organisation suggestions at the micro level play core roles in Chinese STF owners’ reopening decisions. This reasoning is somewhat in line with Vaisey [76], who proposed a dual-process model of culture in action.

### 5.2. Theoretical Implications

This study primarily provides a comprehensive understanding of emerging behavioural decisions faced by STFs amid COVID-19 as they reopen. It also examines and interprets multiple influential factors from a psychological perspective. By proposing and examining an integrated model of PMT and TPB, the study provides a few theoretical and methodological contributions to the literature pertaining to STF risk decisions in tourism management. Despite a variety of tourist risk behaviours described in the existing research, this study adds a new risk type regarding STFs reopening during epidemics from the supply side, which combines occupational health risks and business uncertainty. Regarding the extremely limited focus on STFs in the tourism risk and crisis field, this study contributes to our understanding of STF risk decisions in this new context. More importantly, as one of the first studies to analyse owners’ decision making to understand STF operations, this study provides an alternative theoretical approach for future STF research. The results confirmed the explanatory and predictive power of the model, providing a new theoretical lens for future research on behaviours amid tourist scenarios with substantial external environmental threats. It also shows the theoretical potential for understanding combined health and business risk behaviours by specifying the roles of social processes and mechanisms in discussions regarding the precise relationships of the various dual-process models. Furthermore, the key methodological contribution of this study is the method combination of PLS–SEM and fsQCA, verifying the complementary functions of a symmetrical approach and asymmetrical modelling for an in-depth understanding of complex psychological behaviour, especially in studies integrating theoretical models.

### 5.3. Practical Implications

Various risks and crises always exist, influencing the development of global tourism [9,77]. The empirical findings can be used as a reference for Chinese tourism practitioners to help STFs recover and meet the target supply of policy, finance, etc. First, because STF owners rely on the government, official measures are important to increase owners’ perceived certainty, e.g., transparent and timely information, and their preparedness for the consequences of possible infection. Second, society and collective influence carry the greatest weight, which indicates that the Chinese government can more effectively guide STFs with scientific policies based on epidemic dynamics and market situations. Of course, this recommendation assumes that the government authority has credibility from its earlier, effective anti-epidemic actions. Finally, owners’ self-efficacy has a greater influence on reopening a business than does response efficiency. The STF capabilities should be noted, including the ability not only to reopen during the epidemic but also to resist risks in more typical circumstances. The public sector should first pay attention to increasing STF owners’ ability to reopen, including through financial support, occupational public health protection training, mental health enhancement, etc. Finally, the structural role played by society and politics is an important finding for Chinese STF reopening decision making, but the above implications must also be globally generalised, especially as they pertain to government and public administrators. Globally, the COVID-19 dynamic in many Western high-welfare countries and other neo-liberal developing countries differs from that in China in many aspects. These include the social values of individualism, government policy, and performance on anti-COVID-19 measures and STF financial situations [78]. STF owners’ reopening in other contexts means different kinds or degrees of risk decisions because of the absence of any or all of the following: lockdowns, fear of COVID-19 infection, financial pressure, collective memory of anti-COVID-19 measures, etc.

### 5.4. Limitations and Future Research

Due to limitations during the studied period, we conducted the investigation within a short time and used only the ACPY as a case. The following new theoretical and practical issues are raised for later research. First, why do subjective norms have the greatest impact on behavioural intentions? What are the mechanisms by which these norms work? The various cultural contexts of the different risk scenarios require further verification [75]. Second, a question worth exploring is whether the impact of response efficiency differs based on the relationship between the threat and behaviour. This question requires further systematic exploration regarding risk decisions in the context of multiple threats. Third, the gap between behaviour and intention or attitude [79,80] cannot be neglected; continually tracking and exploring STF business and risk conditions after reopening are required to complete our knowledge of STF resumption and recovery during a public health event.

## Figures and Tables

**Table 1:** Interview participant profiles.

No.	Age	Gender	Business Type	Estimated Losses from COVID-19 (Thousands, RMB Yuan)	Reopened Status (Reopening Date)
1	41	Male	Restaurant	20~30	NO
2	46	Male	Accommodation	40~50	NO
3	32	Male	Traffic service	40~50	NO
4	50	Female	Entertainment	Inestimable	NO
5	29	Female	Travel agencies	10~20	NO
6	32	Female	Restaurant	50~100	YES (27 March)
7	29	Male	Restaurant	≧300	YES (26 March)
8	50	Male	Souvenir shop	100~200	YES (26 March)
9	35	Male	Bar	≧300	NO
10	28	Female	Souvenir shop	≧300	YES (27 March)
11	59	Male	Official for more than 10 years on the ACPY Management Committee working		
12	46	Female	Ticket-taker working in the ACPY for more than ten years		
13	47	Female	Community elite living in the ACPY for 47 years		

**Table 2:** Sample characteristics (N = 265).

Variable	Responses	Frequency	Percent
Gender	Male	129	48.68
	Female	136	51.32
Age	18–25	20	7.55
	26–30	50	18.87
	31–40	102	38.49
	41–50	68	25.66
	Above 50	25	9.43
Business type	Restaurant	84	31.70
	Accommodation	15	5.66
	Souvenir shop	65	24.53
	Travel agencies and tour guides	27	10.18
	Entertainment	9	3.40
	Other	65	24.53
Years in business	0–3	89	33.58
	3–5	62	23.40
	5–10	50	18.87
	10–15	35	13.21
	Above 15	29	10.94
Firm place of business	Leased	223	84.15
	Own	42	15.85
Is STF the only family income source?	Yes	225	84.91
	No	40	15.09

**Table 3:** First-order measurement model and descriptive statistics.

Constructs and Items	Loading	Mean	Skewness	Kurtosis	T Value	CR	AVE
**Behavioural intention to reopen, BIR**						0.952	0.908
I plan to reopen soon (now)	0.955	4.14	−1.296	1.703	135.706		
I intend to reopen soon (now)	0.95	4.08	−1.178	2.345	111.106		
**Perceived infection risk, PIR**						0.883	0.791
Susceptibility	0.931	3.37	−0.422	0.635	39.795		
Severity	0.846	3.76	−0.487	−0.685	18.03		
**Perceived business uncertainty, PBU**						0.932	0.872
The market after reopening is uncertain and risky	0.945	2.86	0.29	0.029	125.269		
Reopening may introduce new difficulties to the business	0.923	2.97	0.262	0.253	75.356		
**Reward**						0.948	0.901
Reopening introduces more business opportunities	0.939	4.28	−1.245	1.447	58.66		
Reopening makes one relaxed and pleasant	0.959	4.26	−1.146	1.355	112.443		
**Response efficacy, RE**						0.89	0.802
Reopening is one of the best ways to relieve the losses resulting from the outbreak	0.915	3.48	0.018	0.073	50.518		
Reopening will help to resolve the backlog	0.875	3.46	−0.034	−0.226	34.176		
**Cost**						0.899	0.817
Epidemic prevention work is difficult and troublesome *	-	3.4	−0.117	−0.401	-		
I need to repeatedly prepare and think about reopening	0.904	3.76	−0.56	0.41	3.682		
It will require much money and energy from me to reopen	0.904	3.57	−0.246	−0.412	3.736		
**Self-efficacy, SE**						0.905	0.76
I am confident that I can reopen well	0.871	3.74	−0.475	0.319	48.904		
It will be easy for me to reopen	0.857	3.35	−0.099	−0.375	38.926		
I can respond to various emergencies in reopening	0.887	3.65	−0.468	0.344	54.368		
**Attitude, Att**						0.878	0.706
Necessary	0.853	4.15	−0.774	0.729	33.918		
Favourable	0.891	4.02	−0.641	0.267	48.98		
Beneficial	0.773	3.73	−0.635	0.071	18.908		
**Subjective norms, SN**						0.906	0.708
Government encourages and advocates for reopening	0.861	3.86	−0.548	0.14	48.731		
Peers may reopen in the near future	0.762	3.63	−0.239	0.181	22.139		
My family and friends support the reopening	0.863	3.7	−0.046	−0.639	50.126		
Experts claim to support reopening and recovery	0.875	3.83	−0.436	0.074	52.399		

* Represents the items deleted in the measurement model test.

**Table 4:** Parameter values of the model fit.

	PMT	TPB	IM	Recommended Threshold
Standardised root mean square residual (SRMR)	0.055	0.069	0.057	<0.08
Goodness-of-fit (GoF)	0.654	0.647	0.708	>0.360
The coefficient of determination (R^2^)	0.512	0.543	0.621	0.67 (substantial), 0.33 (moderate), 0.19 (weak)
The construct cross-validated redundancy (Q^2^)	0.433	0.462	0.525	0 (small), 0.25 (medium), 0.5 (large)

**Table 5:** Model fit test results.

Integrated PMT and TPB Model		Standardised Path Coefficients	Original Model	Standardised Path Coefficients
H1	PIR → BIR	−0.095 *	**PMT model**	
H2	PBU → BIR	−0.225 ***	PIR → BIR	−0.109 *
H3	Rew → BIR	0.145 **	PBU → BIR	−0.291 ***
H4	RE → BIR	0.003 (ns)	Rew → BIR	0.233 ***
H5	Cos → BIR	−0.053 (ns)	RE → BIR	0.080 (ns)
H6	SE → BIR	0.193 ***	Cos → BIR	−0.003 (ns)
H7	Att → BIR	0.183 ***	SE → BIR	0.358 ***
H8	SN → BIR	0.334 ***	**TPB model**	
			Att → BIR	0.215 ***
			SN → BIR	0.402 ***
			SE → BIR	0.286 ***

Path significance: *** *p* < 0.001, ** *p* < 0.01, * *p* < 0.05, and ns *p* > 0.05.

**Table 6:** Necessary conditions analysis.

Condition	H-BIR		L-BIR	
	Consistency	Coverage	Consistency	Coverage
PIR	0.631	0.385	0.415	0.974
~PIR	0.958	0.298	0.738	0.885
PBU	0.668	0.258	0.663	0.988
~PBU	0.969	0.428	0.502	0.853
Rew	0.612	0.688	0.212	0.916
~Rew	0.925	0.234	0.928	0.902
RE	0.878	0.494	0.420	0.911
~RE	0.842	0.274	0.767	0.960
Cos	0.706	0.468	0.378	0.967
~Cos	0.950	0.284	0.792	0.912
SE	0.904	0.567	0.357	0.861
~SE	0.779	0.239	0.821	0.970
Att	0.775	0.677	0.270	0.910
~Att	0.897	0.242	0.904	0.939
SN	0.870	0.641	0.307	0.870
~SN	0.824	0.236	0.874	0.963

**Table 7:** Causal configurations for high and low behavioural intentions.

	PIR	PBU	Rew	RE	Cos	SE	Att	SN	Raw Coverage	Unique Coverage	Consistency
**High Behavioural Intention to Reopen, H-BIR**											
M_H1_	⊗	⊗	●	●		●	●	●	0.505	0.505	0.927
Solution coverage: 0.505; solution consistency: 0.927; consistency cut-off: 0.927											
**Low Behavioural Intention to Reopen, L-BIR**											
M_L1_		●				⊗	⊗	⊗	0.567	0.022	0.995
M_L2_	⊗		⊗		⊗	⊗	⊗	⊗	0.549	0.063	0.992
M_L3_		●		⊗	⊗	⊗	⊗	⊗	0.505	0.005	0.995
M_L4_		●	⊗	⊗	⊗		⊗	⊗	0.508	0.005	0.994
M_L5_	⊗	●			⊗	⊗	⊗	⊗	0.476	0.002	0.994
M_L6_	⊗	●	⊗		⊗		⊗	⊗	0.484	0.005	0.994
M_L7_			⊗	●	●	⊗	⊗	⊗	0.263	0.008	0.991
M_L8_	⊗	●	⊗	⊗	●	⊗		⊗	0.280	0.004	0.994
M_L9_	⊗	⊗	⊗	●		●	⊗	●	0.215	0.007	0.926
M_L10_	⊗	⊗	●	●		●	●	●	0.130	0.001	0.921
M_L11_	⊗	⊗	●	●	⊗	●		●	0.139	0.001	0.908
Solution coverage: 0.707; solution consistency: 0.966; consistency cut-off: 0.920											

Note: Black circles represent the presence of a causal condition, and circles with “×” represent the absence or negation of a causal condition. Blank cells represent irrelevant conditions.

## Data Availability

The data that support the findings of this study are available from the corresponding author upon reasonable request.
